# Supplementary material for: Impact of the COVID-19 pandemic on bone and soft tissue tumor treatment: A single-institution study
Source: PLoS One. 2023 Apr 24;18(4):e0283835. doi: 10.1371/journal.pone.0283835 (PMC10124828; doi:10.1371/journal.pone.0283835)
Supplement: S1 File — (PDF) [file pone.0283835.s001.pdf]

No	Gender	Length of hospital stay	Type of umor	Neoplasm	Grade	Complication	CTCAE	Use of Implant	Hospital cost(yen)	Hospital cost(dollar)
1	M	3	soft tissue	benign		no		no	285480	2854.8
2	F	5	soft tissue	benign		no		no	357080	3570.8
3	M	27	soft tissue	benign		infection	grade2	no	1530030	15300.3
4	F	33	bone	malignant	high	no		no	1125200	11252
5	F	65	soft tissue	benign		sepsis	grade5	no	4505730	45057.3
6	M	9	soft tissue	benign		no		no	523050	5230.5
7	M	5	bone	malignant	high	no		no	379810	3798.1
8	F	18	bone	benign		no		no	1163930	11639.3
9	M	16	bone	malignant	high	no		yes	2113570	21135.7
10	M	40	soft tissue	malignant	high	no		no	2097530	20975.3
11	F	12	bone	malignant	high	no		no	592630	5926.3
12	M	17	soft tissue	benign		no		no	1286780	12867.8
13	M	28	bone	malignant	high	leachate	grade1	yes	5149070	51490.7
14	M	23	soft tissue	malignant	low	no		no	1458950	14589.5
15	F	9	soft tissue	malignant	low	no		no	692640	6926.4
16	F	13	bone	malignant	high	no		no	1375310	13753.1
17	M	89	soft tissue	malignant	high	soft tissue necrosis	grade3	no	3794740	37947.4
18	M	12	bone	malignant	low	no		no	908890	9088.9
19	M	8	soft tissue	malignant	high	soft tissue necrosis	grade3	no	679090	6790.9
20	M	26	soft tissue	malignant	high	soft tissue necrosis	grade3	no	1472520	14725.2
21	F	34	bone	malignant	high	no		yes	5823590	58235.9
22	F	3	soft tissue	malignant	high	delirium	grade1	no	449280	4492.8
23	M	36	bone	malignant	high	electrolyte abnormal	grade2	yes	2587680	25876.8
24	F	52	bone	malignant	high	no		yes	4167060	41670.6
25	M	4	bone	benign		no		no	323630	3236.3
26	M	7	soft tissue	benign		no		no	394020	3940.2
27	M	6	soft tissue	benign		no		no	323910	3239.1
28	M	6	bone	benign		no		no	473370	4733.7
29	F	4	soft tissue	benign		no		no	363010	3630.1
30	M	12	soft tissue	malignant	high	impairment of liver function	grade1	no	1383040	13830.4
31	M	3	soft tissue	malignant	low	no		no	286300	2863
32	F	6	soft tissue	malignant	low	no		no	602440	6024.4
33	M	8	bone	benign		no		no	456260	4562.6
34	F	2	soft tissue	benign		no		no	120800	1208
35	M	21	bone	benign		no		no	969210	9692.1
36	M	7	soft tissue	benign		no		no	443170	4431.7
37	M	9	soft tissue	malignant	high	no		no	692680	6926.8
38	M	24	soft tissue	malignant	low	infection	grade2	no	1147500	11475
39	F	2	soft tissue	benign		no		no	169480	1694.8
40	F	61	soft tissue	malignant	high	infection	grade3	no	3171720	31717.2
41	M	14	bone	benign		no		no	966390	9663.9
42	M	15	bone	benign		no		no	1050480	10504.8
43	F	2	soft tissue	benign		no		no	131410	1314.1
44	M	8	soft tissue	benign		no		no	544840	5448.4
45	F	7	soft tissue	benign		no		no	420230	4202.3
46	M	9	bone	benign		no		no	918870	9188.7
47	M	24	bone	malignant	low	no		no	1309340	13093.4
48	F	32	bone	benign		no		no	1510040	15100.4
49	M	13	soft tissue	benign		no		no	585090	5850.9
50	F	21	soft tissue	malignant	high	no		no	1238630	12386.3
51	F	23	soft tissue	malignant	low	no		no	1115020	11150.2
52	M	15	bone	benign		no		no	621070	6210.7
53	F	11	soft tissue	malignant	high	no		no	514710	5147.1
54	M	9	soft tissue	benign		pneumothorax	grade1	no	469330	4693.3
55	M	21	soft tissue	malignant	high	edema	grade1	no	1169590	11695.9
56	F	11	bone	benign		no		yes	1194680	11946.8
57	M	30	soft tissue	malignant	high	leachate	grade1	no	1247990	12479.9
58	F	9	soft tissue	benign		no		no	409680	4096.8
59	F	8	bone	benign		no		no	520880	5208.8
60	F	6	soft tissue	benign		no		no	328360	3283.6
61	M	8	soft tissue	benign		no		no	387700	3877
62	M	12	bone	malignant	low	no		no	940590	9405.9
63	M	10	bone	benign		no		no	539070	5390.7
64	F	8	soft tissue	benign		no		no	423890	4238.9
65	F	18	bone	benign		no		no	806390	8063.9
66	F	59	soft tissue	malignant	low	soft tissue necrosis	grade3	no	2493640	24936.4
67	F	11	soft tissue	benign		no		no	515730	5157.3
68	M	14	soft tissue	malignant	high	no		yes	1271360	12713.6
69	F	54	soft tissue	malignant	high	no		no	2506930	25069.3
70	F	19	soft tissue	malignant	high	no		no	1141980	11419.8
71	F	7	soft tissue	benign		no		no	421020	4210.2
72	M	20	bone	malignant	low	no		no	955890	9558.9
73	F	10	soft tissue	benign		no		no	475790	4757.9
74	M	11	soft tissue	benign		no		no	562320	5623.2
75	M	11	soft tissue	benign		no		no	1150720	11507.2
76	M	16	bone	malignant	low	no		yes	3043890	30438.9
77	M	14	soft tissue	malignant	high	no		no	856830	8568.3
78	M	29	soft tissue	malignant	high	no		no	1366720	13667.2
79	M	17	bone	malignant	high	hemothorax	grade2	no	1428600	14286
80	M	25	soft tissue	malignant	high	no		no	1088980	10889.8
81	F	6	soft tissue	benign		no		no	394870	3948.7
82	M	20	bone	benign		no		yes	1327720	13277.2
83	M	39	bone	malignant	high	no		yes	3423640	34236.4
84	F	10	bone	benign		no		no	842670	8426.7
85	F	21	soft tissue	malignant	high	no		no	1169590	11695.9
86	M	7	soft tissue	benign		no		no	396250	3962.5
87	F	14	bone	malignant	high	allergy	grade1	yes	2971290	29712.9
88	M	7	soft tissue	benign		no		no	394240	3942.4
89	F	29	soft tissue	malignant	high	no		no	1889820	18898.2
90	M	9	soft tissue	benign		no		no	517570	5175.7
91	F	8	soft tissue	benign		no		no	508170	5081.7
92	M	7	soft tissue	malignant	high	no		no	601050	6010.5
93	F	12	bone	benign		abdominal pain	grade1	no	771920	7719.2
94	F	20	bone	malignant	high	no		yes	2628660	26286.6
95	F	22	bone	malignant	high	no		yes	1987160	19871.6
96	M	4	bone	malignant	high	no		no	426030	4260.3
97	F	7	soft tissue	benign		no		no	355980	3559.8
98	M	35	soft tissue	malignant	high	no		no	1985330	19853.3
99	M	8	soft tissue	benign		no		no	463420	4634.2
100	M	11	soft tissue	benign		no		no	553570	5535.7
101	M	58	soft tissue	malignant	high	soft tissue necrosis	grade3	no	2321590	23215.9
102	M	36	bone	malignant	high	no		yes	5561680	55616.8
103	M	15	bone	malignant	high	no		yes	1042080	10420.8
104	F	14	bone	benign		no		no	696250	6962.5
105	M	27	bone	benign		allergy	grade1	yes	1384530	13845.3
106	M	7	soft tissue	benign		no		no	350250	3502.5
107	M	18	bone	benign		no		no	841120	8411.2
108	M	8	soft tissue	malignant		no		no	547200	5472
109	F	6	soft tissue	benign		no		no	367630	3676.3
110	M	11	soft tissue	malignant	high	wound necrosis	grade2	no	658710	6587.1
111	F	29	soft tissue	malignant	high	no		no	1420110	14201.1
112	F	15	bone	malignant	high	no		no	2938840	29388.4
113	M	11	bone	benign		no		no	540420	5404.2
114	F	35	soft tissue	malignant	high	pneumothorax	grade3	no	1593830	15938.3

No	Age	Gender	Length of hospital stay	Type of tumor	Neoplasm	grade	Complication	CTCAE	Operation Time	Use of Implant	Hospital cost(yen)	Hospital cost(dollar)
1	19	M	28	bone	malignant	high	no		4:38	yes	3683600	36836
2	72	F	2	soft tissue	benign		no		0:20	no	131410	1314.1
3	71	M	7	soft tissue	benign		no		0:45	no	400760	4007.6
4	19	F	117	bone	benign		fracture	grade3	2:24	no	4692620	46926.2
5	88	M	71	soft tissue	malignant	high	lymph leakage	grade1	5:49	no	3900770	39007.7
6	71	F	3	bone	malignant	high	no		0:28	no	250490	2504.9
7	64	M	5	soft tissue	malignant	high	no		0:49	no	530520	5305.2
8	72	F	11	soft tissue	malignant	high	no		0:30	no	703870	7038.7
9	30	F	7	soft tissue	benign		no		0:25	no	371650	3716.5
10	76	M	8	bone	benign		no		0:16	no	450870	4508.7
11	42	F	16	soft tissue	malignant	high	no		1:10	no	805530	8055.3
12	19	M	6	bone	benign		no		0:29	no	396650	3966.5
13	57	F	10	bone	benign		no		1:17	no	620300	6203
14	56	M	3	soft tissue	benign		no		0:22	no	274720	2747.2
15	38	F	10	bone	benign		no		3:53	no	602160	6021.6
16	69	F	13	bone	benign		no		1:40	yes	941710	9417.1
17	18	M	7	bone	benign		no		1:54	no	787880	7878.8
18	20	M	11	bone	benign		paralysis	grade2	2:55	no	665440	6654.4
19	84	F	8	soft tissue	benign		no		0:15	no	349910	3499.1
20	75	F	12	bone	benign		no		1:22	no	772330	7723.3
21	40	M	7	bone	malignant	high	no		1:26	yes	715680	7156.8
22	63	F	8	bone	benign		no		0:44	no	444770	4447.7
23	5	M	6	soft tissue	malignant	low	no		0:49	no	429720	4297.2
24	20	M	8	soft tissue	malignant	high	no		0:33	no	619030	6190.3
25	70	F	8	bone	malignant	high	no		0:35	no	431730	4317.3
26	83	F	11	soft tissue	benign		no		0:40	no	517930	5179.3
27	19	M	7	bone	benign		no		0:49	no	408890	4088.9
28	68	F	5	soft tissue	benign		no		0:39	no	334370	3343.7
29	13	M	5	bone	benign		no		0:45	no	461900	4619
30	44	F	7	soft tissue	benign		no		0:34	no	498800	4988
31	34	M	6	bone	benign		no		0:31	no	340540	3405.4
32	54	F	9	bone	benign		no		1:13	no	603430	6034.3
33	48	M	16	bone	benign		no		1:06	no	882330	8823.3
34	49	M	3	soft tissue	benign		no		0:33	no	242190	2421.9
35	56	M	3	soft tissue	malignant	high	no		0:59	no	367010	3670.1
36	38	F	26	soft tissue	benign		hypoalbuminemia	grade2	1:20	no	958550	9585.5
37	27	M	8	bone	benign		no		0:41	no	532200	5322
38	42	F	16	soft tissue	malignant	high	no		2:12	no	1015840	10158.4
39	77	M	31	soft tissue	malignant	high	Urinary tract infections	grade2	3:23	no	1364890	13648.9
40	44	F	3	soft tissue	benign		no		0:19	no	260430	2604.3
41	28	F	32	bone	benign		no		3:13	no	1688240	16882.4
42	81	F	22	bone	malignant	high	no		1:21	yes	1142660	11426.6
43	67	M	8	soft tissue	benign		no		0:55	no	448530	4485.3
44	66	M	5	soft tissue	benign		no		0:21	no	362520	3625.2
45	41	F	8	bone	benign		no		0:29	no	547100	5471
46	59	F	15	bone	benign		no		0:59	no	830190	8301.9
47	8	M	3	soft tissue	benign		no		0:17	no	286540	2865.4
48	88	M	88	soft tissue	benign		wound infection	grade3	1:27	no	2733080	27330.8
49	63	F	8	soft tissue	benign		no		1:44	no	736260	7362.6
50	74	M	21	bone	benign		no		1:38	no	1320640	13206.4
51	73	M	8	soft tissue	benign		no		1:12	no	509470	5094.7
52	39	F	14	soft tissue	benign		no		2:03	no	812150	8121.5
53	58	M	5	bone	malignant	high	no		1:33	yes	817420	8174.2
54	55	M	5	bone	malignant		no		0:49	no	624870	6248.7
55	8	M	5	bone	benign		no		1:00	no	587670	5876.7
56	59	M	7	soft tissue	malignant	high	no		0:42	no	599130	5991.3
57	20	M	11	soft tissue	malignant	high	no		1:02	no	582280	5822.8
58	32	F	6	bone	benign		no		0:24	no	347210	3472.1
59	81	F	6	soft tissue	benign		no		1:20	no	400230	4002.3
60	40	M	18	bone	malignant	high	rash	grade2	3:20	yes	3523630	35236.3
61	72	M	8	soft tissue	benign		no		0:32	no	405040	4050.4
62	39	F	8	soft tissue	benign		no		0:16	no	413430	4134.3
63	29	M	8	soft tissue	benign		no		0:23	no	435240	4352.4
64	5	M	28	bone	benign		fracture	grade1	1:02	no	1919510	19195.1
65	78	M	67	soft tissue	malignant	high	soft tissue necrosis	grade3	8:35	no	3258760	32587.6
66	47	M	16	soft tissue	malignant	high	nausea	grade1	0:32	no	1013560	10135.6
67	77	M	8	bone	benign		no		1:10	no	527320	5273.2
68	77	F	22	bone	malignant	high	dehydration	grade2	2:34	no	1502310	15023.1
69	40	M	14	bone	benign		no		1:06	no	932230	9322.3
70	26	M	46	soft tissue	malignant	high	no		3:50	yes	5943380	59433.8
71	43	F	26	bone	benign		no		2:03	yes	1387680	13876.8
72	69	F	4	bone	benign		no		0:39	no	341170	3411.7
73	71	F	8	soft tissue	benign		swelling	grade1	0:47	no	409660	4096.6
74	22	M	3	soft tissue	benign		no		0:23	no	241370	2413.7
75	12	F	6	bone	benign		no		2:00	no	699890	6998.9
76	38	F	4	bone	benign		no		0:46	no	367030	3670.3
77	30	M	3	soft tissue	benign		no		0:24	no	256340	2563.4
78	57	M	6	soft tissue	benign		no		0:46	no	358530	3585.3
79	55	F	9	soft tissue	benign		no		0:54	no	442060	4420.6
80	61	F	6	soft tissue	benign		no		0:39	no	358900	3589
81	66	M	24	soft tissue	malignant	high	no		2:18	no	1235190	12351.9
82	52	M	14	bone	benign		no		1:15	no	689800	6898
83	69	F	8	soft tissue	benign		no		0:45	no	416050	4160.5
84	76	M	14	soft tissue	malignant	high	no		1:08	no	841750	8417.5
85	20	F	9	bone	benign		no		1:15	no	612790	6127.9
86	53	F	6	bone	malignant	high	no		0:31	no	371210	3712.1
87	73	F	5	soft tissue	benign		no		0:38	no	325080	3250.8
88	73	F	5	soft tissue	benign		no		0:31	no	345350	3453.5
89	66	M	3	bone	benign		no		1:26	no	287210	2872.1
90	69	M	16	soft tissue	malignant	low	hematoma	grade1	1:12	no	104651	1046.51
91	70	M	5	soft tissue	benign		no		0:32	no	290120	2901.2
92	43	M	4	soft tissue	benign		no		0:25	no	299460	2994.6
93	53	F	6	soft tissue	benign		no		1:05	no	596650	5966.5
94	55	M	4	soft tissue	benign		no		0:52	no	319020	3190.2
95	47	M	66	soft tissue	malignant	high	leachate	grade1	4:23	no	2250140	22501.4
96	35	F	3	soft tissue	malignant	high	no		0:30	no	242760	2427.6
97	53	F	31	bone	malignant	high	soft tissue necrosis	grade3	5:45	yes	3745080	37450.8
98	42	F	43	bone	malignant	high	leachate	grade2	1:49	no	789510	7895.1
99	28	M	29	soft tissue	benign		no		5:36	yes	1594590	15945.9
100	69	M	35	soft tissue	malignant	high	no		8:14	no	2037890	20378.9
101	64	M	5	soft tissue	malignant	high	no		1:09	no	540860	5408.6
102	81	M	16	soft tissue	malignant	low	no		2:08	no	957130	9571.3
103	37	M	12	bone	malignant		no		2:28	no	1073620	10736.2
104	18	F	5	soft tissue	benign		no		0:35	no	351610	3516.1
105	79	F	9	soft tissue	malignant	high	no		0:58	no	664480	6644.8
106	69	M	23	bone	malignant	high	no		1:40	yes	1706160	17061.6
107	39	M	6	bone	malignant	high	no		0:46	no	305700	3057
108	50	F	18	soft tissue	malignant	high	no		2:01	no	1007590	10075.9
109	80	F	7	soft tissue	benign		no		0:24	no	407780	4077.8
110	59	M	4	soft tissue	benign		no		0:49	no	390980	3909.8
111	47	M	4	soft tissue	benign		no		0:34	no	331770	3317.7
112	77	M	3	soft tissue	benign		no		0:56	no	277390	2773.9
113	79	M	16	soft tissue	benign		no		0:29	no	585120	5851.2
114	78	M	23	soft tissue	malignant	high	leachate	grade2	2:07	no	670070	6700.7
115	31	F	10	bone	malignant		no		1:28	no	592190	5921.9
116	56	F	7	soft tissue	benign		no		1:04	no	395160	3951.6
117	25	F	4	soft tissue	benign		no		0:20	no	314340	3143.4
118	65	F	6	soft tissue	benign		no		1:35	no	378840	3788.4
119	60	M	25	bone	malignant	high	no		2:02	yes	3716630	37166.3
120	62	F	3	soft tissue	benign		no		0:17	no	280760	2807.6
121	85	F	8	soft tissue	benign		no		0:28	no	421660	4216.6
122	73	M	10	soft tissue	malignant	high	skin necrosis	grade2	0:23	no	495670	4956.7
123	67	F	3	soft tissue	malignant	high	no		0:22	no	230840	2308.4
124	53	M	29	soft tissue	malignant	high	blister formation	grade1	3:02	no	1590900	15909
125	39	M	26	bone	malignant	high	no		3:45	yes	3886660	38866.6
126	35	F	5	soft tissue	malignant	high	phantom pain	grade1	0:50	no	402300	4023
127	53	M	11	bone	benign		no		2:30	yes	1012830	10128.3
128	34	M	13	soft tissue	benign		no		0:31	no	551420	5514.2
129	48	F	4	bone	benign		no		0:43	no	310200	3102
130	67	M	3	soft tissue	benign		no		0:39	no	294390	2943.9
131	80	M	28	soft tissue	malignant	low	no		3:40	no	1361600	13616
132	53	M	3	bone	benign		no		0:44	no	275210	2752.1
133	74	M	26	bone	malignant	high	no		2:21	yes	1893590	18935.9
134	62	M	8	bone	benign		no		0:55	no	421690	4216.9
135	81	M	51	soft tissue	benign		no		1:22	no	2534080	25340.8
136	11	F	3	bone	benign		no		0:40	no	387580	3875.8
137	16	M	5	bone	benign		no		0:31	no	324690	3246.9
138	67	F	23	bone	malignant	high	no		1:13	yes	1263740	12637.4
139	20	M	6	bone	benign		no		1:09	no	354400	3544

No	Gender	Type of tumor	Neoplasm
1	M	soft tissue	benign
2	F	soft tissue	malignant
3	F	soft tissue	benign
4	F	soft tissue	malignant
5	F	soft tissue	benign
6	F	soft tissue	benign
7	M	soft tissue	benign
8	F	soft tissue	benign
9	F	soft tissue	malignant
10	M	soft tissue	benign
11	M	soft tissue	benign
12	M	soft tissue	benign
13	F	soft tissue	benign
14	F	soft tissue	benign
15	F	soft tissue	benign
16	F	soft tissue	benign

No	Gender	Type of tumor	Neoplasm
1	F	soft tissue	benign
2	F	soft tissue	benign
3	M	soft tissue	benign
4	F	soft tissue	benign
5	M	soft tissue	benign
6	M	soft tissue	benign
7	M	soft tissue	benign
8	F	soft tissue	benign
9	F	soft tissue	benign
10	M	soft tissue	benign
11	F	soft tissue	benign
12	F	soft tissue	benign
13	M	soft tissue	benign
14	F	soft tissue	benign
15	F	soft tissue	benign
16	M	soft tissue	benign
17	M	soft tissue	benign
18	F	soft tissue	benign
19	M	soft tissue	malignant
20	M	soft tissue	benign
21	F	soft tissue	benign
22	F	soft tissue	benign
23	M	soft tissue	benign
24	F	soft tissue	benign
25	F	soft tissue	malignant
26	M	soft tissue	malignant

No	Gender	Age	Diagnosis	Chemotherapy	Complication	CTCAE
1	M	51	dedifferentiated liposarcoma	Trabectedin	impairment of liver function	grade1
2	M	52	dedifferentiated liposarcoma	Trabectedin	impairment of liver function	grade1
3	F	48	synovial sarcoma	DOX、IFM	nausea	grade2
4	M	52	dedifferentiated liposarcoma	Trabectedin	impairment of liver function	grade1
5	M	27	myoepithelial carcinoma	IE	no	
6	M	16	osteosarcoma	VDC	no	
7	F	15	synovial sarcoma	DOX、IFM	Neutrophil count decreased	grade3
8	M	39	osteosarcoma	DOX　CDDP	no	
9	F	22	mesenchymal chondrosarcoma	VDC	nausea	grade1
10	M	67	undifferentiated pleomorphic sarcoma	DOX	no	
11	F	23	osteosarcoma	VDC	no	
12	M	51	pleomorphic rhabdmyosarcoma	Trabectedin	no	
13	M	51	pleomorphic rhabdmyosarcoma	Trabectedin	Febrile neutropenia	grade4
14	M	27	myxoid liposarcoma	DOX　CDDP	diarrhea	grade1
15	F	23	osteosarcoma	VDC	Neutrophil count decreased	grade4
16	F	23	osteosarcoma	VDC	Neutrophil count decreased	grade4
17	M	39	osteosarcoma	IFM	no	
18	M	69	myxoid liposarcoma	Trabectedin	nausea	grade1
19	F	22	mesenchymal chondrosarcoma	IE	no	
20	M	39	osteosarcoma	IFM	no	
21	M	69	myxoid liposarcoma	Trabectedin	nausea	grade1
22	M	21	rhabdmyosarcoma	DOX、IFM	nausea	grade1
23	F	15	synovial sarcoma	DOX、IFM	nausea	grade1
24	M	51	pleomorphic rhabdmyosarcoma	Trabectedin	Neutrophil count decreased	grade3
25	F	15	synovial sarcoma	DOX、IFM	nausea	grade1
26	F	22	mesenchymal chondrosarcoma	IE	no	
27	M	63	myxoid liposarcoma	Trabectedin	no	
28	M	27	myoepithelial carcinoma	IE	no	
29	F	15	synovial sarcoma	DOX、IFM	no	
30	M	77	osteosarcoma	GEM DTX	no	
31	M	16	osteosarcoma	VDC	no	
32	M	69	myxoid liposarcoma	Trabectedin	nausea	grade1
33	M	27	myxoid liposarcoma	DOX　CDDP	nausea	grade1
34	M	39	osteosarcoma	IFM	no	
35	M	27	myoepithelial carcinoma	IE	no	
36	F	22	mesenchymal chondrosarcoma	IE	no	
37	M	39	osteosarcoma	DOX　CDDP	no	
38	M	27	myxoid liposarcoma	DOX　CDDP	no	
39	F	22	mesenchymal chondrosarcoma	Trabectedin	Neutrophil count decreased	grade4
40	F	23	osteosarcoma	VDC	Pneumothorax	grade1
41	M	21	rhabdmyosarcoma	DOX、IFM	nausea	grade1
42	F	23	osteosarcoma	VDC	no	
43	F	15	synovial sarcoma	DOX、IFM	Neutrophil count decreased	grade3
44	M	39	osteosarcoma	DOX　CDDP	no	
45	M	36	extraskeltal ewing sarcoma	IE	no	
46	F	16	osteosarcoma	DOX　CDDP	no	
47	M	36	extraskeltal ewing sarcoma	VDC	no	
48	M	55	dedifferentiated liposarcoma	ICE	no	
49	F	47	leiomyosarcoma	ICE	Neutrophil count decreased	grade2
50	M	36	extraskeltal ewing sarcoma	IE	no	
51	M	69	undifferentiated pleomorphic sarcoma	DOX、IFM	no	
52	F	16	osteosarcoma	MTX	no	
53	M	36	extraskeltal ewing sarcoma	VDC	no	
54	F	66	myxofibrosarcoma	Trabectedin	no	
55	F	16	osteosarcoma	MTX	nausea	grade1
56	F	16	osteosarcoma	DOX　CDDP	Neutrophil count decreased	grade4
57	F	16	osteosarcoma	ICE	nausea	grade1
58	M	36	extraskeltal ewing sarcoma	IE	no	
59	M	36	extraskeltal ewing sarcoma	VDC	no	
60	M	55	dedifferentiated liposarcoma	ICE	Platelet count decreased	grade3
61	M	36	extraskeltal ewing sarcoma	VDC	Neutrophil count decreased	grade4
62	M	36	extraskeltal ewing sarcoma	IE	nausea	grade1
63	F	54	undifferentiated pleomorphic sarcoma	DOX、IFM	no	
64	F	54	undifferentiated pleomorphic sarcoma	DOX、IFM	no	
65	M	69	undifferentiated pleomorphic sarcoma	DOX、IFM	no	
66	M	69	undifferentiated pleomorphic sarcoma	DOX、IFM	platelet count decreased	grade 1
67	M	27	myoepithelial carcinoma	IE	no	
68	M	27	myoepithelial carcinoma	IE	no	
69	M	63	myxoid liposarcoma	Trabectedin	no	
70	M	63	myxoid liposarcoma	Trabectedin	no	
71	M	63	myxoid liposarcoma	Trabectedin	no	
72	F	22	mesenchymal chondrosarcoma	VDC	Neutrophil count decreased	grade4
73	F	67	fibromyxoidsarcoma	Trabectedin	no	
74	M	27	myxoid liposarcoma	DOX　CDDP	no	
75	M	27	myxoid liposarcoma	DOX　CDDP	no	
76	M	27	myxoid liposarcoma	Trabectedin	impairment of liver function	grade1
77	M	27	myxoid liposarcoma	Trabectedin	no	
78	M	41	myxoid liposarcoma	DOX、IFM	no	
79	M	41	myxoid liposarcoma	DOX、IFM	no	
80	M	41	myxoid liposarcoma	DOX、IFM	no	
81	M	41	myxoid liposarcoma	DOX、IFM	no	
82	M	64	leiomyosarcoma	DOX	no	
83	M	41	osteosarcoma	IFM	no	
84	M	52	myxoid liposarcoma	Trabectedin	no	
85	M	52	myxoid liposarcoma	Trabectedin	no	
86	M	52	myxoid liposarcoma	Trabectedin	no	
87	M	68	undifferentiated pleomorphic sarcoma	DOX	no	
88	M	68	undifferentiated pleomorphic sarcoma	Eribulin	Neutrophil count decreased	grade4
89	M	64	undifferentiated pleomorphic sarcoma	DOX、IFM	Heart failure	grade3
90	M	19	osteosarcoma	DOX　CDDP	Febrile neutropenia	grade2

No	Gender	Age	Diagnosis	Chemotherapy	Complication	CTCAE
1	M	57	leiomyosarcoma	DOX、IFM	Febrile neutropenia	grade3
2	F	15	osteosarcoma	Eribulin	no	
3	M	26	myxoid liposarcoma	DOX、IFM	no	
4	M	26	myoepithelial carcinoma	IE	fever	grade1
5	F	42	synovial sarcoma	DOX、IFM	Neutrophil count decreased	grade4
6	F	22	mesenchymal chondrosarcoma	Trabectedin	no	
7	M	51	pleomorphic rhabdmyosarcoma	Trabectedin	Febrile neutropenia	grade3
8	M	26	myxoid liposarcoma	DOX、IFM	no	
9	M	60	rhabdmyosarcoma	DOX	no	
10	F	22	mesenchymal chondrosarcoma	IE	no	
11	F	42	synovial sarcoma	DOX、IFM	Neutrophil count decreased	grade4
12	F	15	osteosarcoma	Eribulin	Neutrophil count decreased	grade4
13	F	22	mesenchymal chondrosarcoma	Trabectedin	no	
14	M	51	pleomorphic rhabdmyosarcoma	Eribulin	Neutrophil count decreased	grade4
15	F	22	mesenchymal chondrosarcoma	IE	no	
16	M	51	pleomorphic rhabdmyosarcoma	IFM	no	
17	F	42	synovial sarcoma	DOX、IFM	Neutrophil count decreased	grade3
18	M	26	myoepithelial carcinoma	IE	nausea	grade1
19	F	15	osteosarcoma	DOX	no	
20	M	51	pleomorphic rhabdmyosarcoma	IFM	platelet count decreased	grade3
21	F	15	osteosarcoma	GEM DTX	Neutrophil count decreased	grade3
22	M	51	pleomorphic rhabdmyosarcoma	VDC	no	
23	F	22	mesenchymal chondrosarcoma	Trabectedin	no	
24	F	47	osteosarcoma	IFM	no	
25	M	19	osteosarcoma	IFM	Pneumothorax	grade3
26	M	47	extraskeltal ewing sarcoma	ICE	no	
27	F	46	leiomyosarcoma	Trabectedin	Neutrophil count decreased	grade3
28	M	19	osteosarcoma	MTX	no	
29	M	16	ewing sarcoma	VDC	Neutrophil count decreased	grade4
30	M	47	extraskeltal ewing sarcoma	DOX、IFM	platelet count decreased	grade4
31	M	47	synovial sarcoma	DOX、IFM	nausea	grade1
32	M	19	osteosarcoma	GEM DTX	sseminated intravascular coagula	grade5
33	F	49	pleomorphic rhabdmyosarcoma	DOX、IFM	no	
34	M	19	osteosarcoma	GEM DTX	Pleural effusion	grade3
35	M	19	osteosarcoma	IFM	no	
36	F	49	pleomorphic rhabdmyosarcoma	DOX、IFM	no	
37	F	46	leiomyosarcoma	Trabectedin	Neutrophil count decreased	grade3
38	F	46	leiomyosarcoma	Trabectedin	nausea	grade1
39	M	19	osteosarcoma	DOX　CDDP	no	
40	M	37	epithelioid sarcoma	MTX	no	
41	M	47	synovial sarcoma	DOX、IFM	nausea	grade1
42	M	47	extraskeltal ewing sarcoma	ICE	no	
43	F	46	leiomyosarcoma	Trabectedin	no	
44	M	37	epithelioid sarcoma	MTX	no	
45	M	37	epithelioid sarcoma	MTX	no	
46	F	46	leiomyosarcoma	VDC	no	
47	F	46	leiomyosarcoma	VDC	Neutrophil count decreased	grade4
48	F	46	leiomyosarcoma	VDC	no	
49	M	26	myoepithelial carcinoma	IE	no	
50	M	26	myoepithelial carcinoma	IE	no	
51	M	50	pleomorphic rhabdmyosarcoma	VDC	no	
52	M	50	pleomorphic rhabdmyosarcoma	GEM DTX	no	
53	M	50	pleomorphic rhabdmyosarcoma	GEM DTX	no	
54	F	22	mesenchymal chondrosarcoma	Trabectedin	no	
55	F	22	mesenchymal chondrosarcoma	Trabectedin	no	
56	F	22	mesenchymal chondrosarcoma	Trabectedin	Neutrophil count decreased	grade3
57	F	22	mesenchymal chondrosarcoma	IE	no	
58	F	22	mesenchymal chondrosarcoma	Trabectedin	no	
59	M	58	leiomyosarcoma	GEM DTX	no	
60	M	47	synovial sarcoma	DOX、IFM	no	
61	M	47	synovial sarcoma	DOX、IFM	no	
62	M	47	synovial sarcoma	DOX、IFM	Neutrophil count decreased	grade4
63	M	69	malignant melanoma	DOX	no	
64	F	16	osteosarcoma	IFM	Neutrophil count decreased	grade3
