# Supplementary material for: Impact of the COVID-19 pandemic on bone and soft tissue tumor treatment: A single-institution study
Source: PLoS One. 2023 Apr 24;18(4):e0283835. doi: 10.1371/journal.pone.0283835 (PMC10124828; doi:10.1371/journal.pone.0283835)
Supplement: Attachment — Submitted filename: Response_to_Reviewers.docx [file pone.0283835.s002.docx]

**Journal Requirements:**

When submitting your revision, we need you to address these additional requirements.

1. Please ensure that your manuscript meets PLOS ONE's style requirements, including those for file naming. The PLOS ONE style templates can be found at 
<https://journals.plos.org/plosone/s/file?id=wjVg/PLOSOne_formatting_sample_main_body.pdf> and 
<https://journals.plos.org/plosone/s/file?id=ba62/PLOSOne_formatting_sample_title_authors_affiliations.pdf>.

**Response**: We thank the editor for these suggestions. We have gone through these templates and ensured that the style requirements of PLOS ONE are met by making appropriate stylistic corrections in the revised manuscript.

2. Please provide additional details regarding participant consent. In the ethics statement in the Methods and online submission information, please ensure that you have specified (1) whether consent was informed and (2) what type you obtained (for instance, written or verbal, and if verbal, how it was documented and witnessed). If your study included minors, state whether you obtained consent from parents or guardians. If the need for consent was waived by the ethics committee, please include this information.

If you are reporting a retrospective study of medical records or archived samples, please ensure that you have discussed whether all data were fully anonymized before you accessed them and/or whether the IRB or ethics committee waived the requirement for informed consent. If patients provided informed written consent to have data from their medical records used in research, please include this information.

**Response**: We thank the editor for these suggestions. Our research was a retrospective study, which was approved by the Institutional Review Board of Osaka City University Graduate School of Medicine. It was performed in accordance with the ethical standards laid down in the Declaration of Helsinki (no. 4394). The study participants provided informed consent prior to their inclusion in the study. This information has been included in the methods section of the manuscript (Lines 113-116).

Lines 113-116: This retrospective study was approved by the Institutional Review Board of Osaka City University Graduate School of Medicine and was performed in accordance with the ethical standards laid down in the Declaration of Helsinki (no. 4934). The subjects included in the study provided informed consent prior to their inclusion in the study.

3. Please include captions for your Supporting Information files at the end of your manuscript, and update any in-text citations to match accordingly. Please see our Supporting Information guidelines for more information: <http://journals.plos.org/plosone/s/supporting-information>.

**Response**: We thank the editor for these insights. We have added the captions for our Supporting Information files at the end of the manuscript (lines 367-374).

4. Please review your reference list to ensure that it is complete and correct. If you have cited papers that have been retracted, please include the rationale for doing so in the manuscript text, or remove these references and replace them with relevant current references. Any changes to the reference list should be mentioned in the rebuttal letter that accompanies your revised manuscript. If you need to cite a retracted article, indicate the article’s retracted status in the References list and also include a citation and full reference for the retraction notice.

**Response**: We thank the editor for the advice. We have rechecked our reference list and ensured that it is complete and conforms to the journal's requirements.

**Review Comments to the Author**

 Reviewer #1: The manuscript entitled "Impact of the COVID-19 pandemic on bone and soft tissue tumor treatment: A single-institution study" by Yoshitaka Ban and co-authors addresses a common and widely-known problem of COVID-19 pandemic and patient treatments. Here, the authors retrospectively analyzed their clinical performance regarding oncological therapy of patients suffering from bone and soft tissue tumors. The authors could show that there were mainly no differences in treatment during COVID-19 compared to no-Covid-19 period except that significantly more patients have been operated during hospitalization. Furthermore the authors show that length of hospital stay was longer and hospital costs were higher.
The manuscript is well written and structured, however, there are some issues that should be a addressed before a recommendation for publication can be given:

1. Please show the results in table 1 that more patients have been operated during hospitalization during COVID-19 pandemic.

**Response**: We thank the reviewer for evaluating our manuscript and for this valuable suggestion. The number of surgeries for malignant bone and soft tissue tumors in group C (n=57) tended to be higher compared to that in group NC (n=50). Table 1 shows the comparison of the number of inpatients between group C (n=114) and group NC (n=139). There was lower number of inpatients in group C. Nevertheless, the number of surgeries for malignant bone and soft tissue tumors in group C was higher and was not affected by the COVID-19 pandemic. We have added the following statement to the results section.

Line 137-139： The number of inpatients in group C was higher than that in group NC, while the number of surgeries for malignant bone and soft tissue tumors was higher than in group NC (P=0.01).

2. Please comment on this point why more patients have been operated during hospitalization!

**Response**: We thank the reviewer for evaluating our manuscript and for this insightful suggestion. At our hospital, outpatient surgery is performed under local anesthesia when the tumor is small and subcutaneous. In all other cases, however, patients are hospitalized prior to surgery, and surgery is performed under general anesthesia after confirming that the patient is in good general condition.

3. Are the differences of LOS and costs are the result that more patients have been operated during hospitalization? Please comment on that.

**Response**: We thank the reviewer for evaluating our manuscript and for these insightful comments. As suggested, this may be partly due to the fact that many patients underwent surgery during their hospitalization. Before the coronavirus pandemic, patients were often hospitalized the day before surgery. During the coronavirus pandemic, however, patients were hospitalized several days before surgery to confirm the absence of fever and respiratory symptoms, and PCR testing was also performed on the day of admission. Therefore, the need to conduct PCR tests and chest CT after the coronavirus outbreak to rule out pneumonia may have been a cause of the increased hospital costs and prolonged length of stay.

Line 231-237: patients were often hospitalized the day before surgery before the coronavirus pandemic. During the COVID-19 pandemic, however, patients were hospitalized several days before surgery to confirm the absence of fever and respiratory symptoms, and PCR testing was also performed on the day of admission. Therefore, the need to conduct PCR tests and chest computed tomography after the coronavirus outbreak to rule out pneumonia may have been a cause for the increased hospital costs and prolonged length of stay.

4. Please add more actual and important references such as PMID: 34624250, PMID: 33021869.

**Response**: We thank the reviewer for their advice. We believe that citing these papers has indeed improved our manuscript. We have cited these articles in lines 194-196, 253-256, and 258-265.

Lines 194-196: Several reports have shown that the COVID-19 pandemic has contributed to a decrease in the number of surgeries for various carcinomas [18,20,21]

Lines 253-256: The cancer surgery system worldwide has been found to be fragile to lockdowns [18], and it is important to better monitor changes in the quality of care, identify the causes of these changes, and take a variety of measures accordingly since these global healthcare crises are expected to occur in the future.

Lines 258-265: In addition, there are reports that the complications of postoperative pneumonia were reduced by creating a dedicated COVID-19-free pathway in the operating room, ward, and intensive care unit [27]. In our hospital, patients who have developed COVID-19 are transported separately from the normal route, and an isolation ward is set up in our hospital. In addition, as shown in Fig. 1, we respond to such medical crises by making full use of information technology during surgery to minimize the number of people coming in and out of the hospital as much as possible.

References:

18.	collaborative COVID, Ademuyiwa A, Adisa A, AlAmeer E, Arnaud AP, Ayasra F, et al. Effect of COVID-19 pandemic lockdowns on planned cancer surgery for 15 tumour types in 61 countries: an international, prospective, cohort study. Lancet Oncol. 2021;22: 1507-1517. doi: [10.1016/S1470-2045(21)00493-9](https://doi.org/10.1016/s1470-2045(21)00493-9).

27.	Glasbey JC, Nepogodiev D, Simoes JFF, Omar O, Li E, Venn ML, et al. Elective cancer surgery in COVID-19–Free Surgical Pathways during the SARS-CoV-2 pandemic: an international, multicenter, comparative cohort study. J Clin Oncol. 2021;39: 66-78. doi: [10.1200/JCO.20.01933](https://doi.org/10.1200/jco.20.01933).

Reviewer #2: The article is interesting, well written and analyzes the management problem of rare tumors during the COVID-19 pandemic

I would just suggest that you improved the discussion by comparing your data with other single-center experiences or other reports.

Please consider these papers to spice up the discussion:
<https://doi.org/10.1016/j.suronc.2022.101859>
10.7759/cureus.30785
<https://doi.org/10.3390/healthcare10112329>
<https://doi.org/10.1186/s12913-022-08166-0>
<https://doi.org/10.1002/jso.26256>

**Response**: We thank the reviewer for their advice. We believe that citing these papers has indeed improved our manuscript. We have cited these articles in lines 189-190, 194-198, 256-258, and 260-265.

Lines 189-190: the pandemic has also affected the surgical field [18–21] and caused the postponement of scheduled surgeries [14].

Lines 194-198: Several reports have shown that the COVID-19 pandemic has contributed to a decrease in the number of surgeries for various carcinomas [18,20,21]; conversely, other studies have reported that the number of tumor surgeries has increased [22], and the impact of the COVID-19 pandemic on surgery is controversial.

Lines 256-258: Previous reports have shown a minimal impact of COVID-19, largely due to the decision to promptly halt all elective surgical procedures and deferrable outpatient care [26].

Lines 260-265: In our hospital, patients who have developed COVID-19 are transported separately from the normal route, and an isolation ward is set up in our hospital. In addition, as shown in Fig. 1, we respond to such medical crises by making full use of information technology during surgery to minimize the number of people coming in and out of the hospital as much as possible.

References:

19.	Carvalho AS, Brito Fernandes Ó, de Lange M, Lingsma H, Klazinga N, Kringos D. Changes in the quality of cancer care as assessed through performance indicators during the first wave of the COVID-19 pandemic in 2020: a scoping review. BMC Health Serv Res. 2022;22: 786. doi: [10.1186/s12913-022-08166-0](https://doi.org/10.1186/s12913-022-08166-0).

20.	Di Martino G, Cedrone F, Di Giovanni P, Romano F, Staniscia T. Impact of COVID-19 pandemic on oncological surgery activities: a retrospective study from a Southern Italian Region. Healthcare (Basel). 2022;10: 2329. doi: [10.3390/healthcare10112329](https://doi.org/10.3390/healthcare10112329).

21.	de Bock ED, Herman ES, Bastian OW, Filipe MD, Vriens MR, Richir MC. Systematic review and meta-analysis determining the effect of implemented COVID-19 guidelines on surgical oncology volumes and clinical outcomes. Surg Oncol. 2022;45: 101859. doi: [10.1016/j.suronc.2022.101859](https://doi.org/10.1016/j.suronc.2022.101859). [Epub 2022 Oct 4].

22.	Mylonakis A, Kalfoutzou A, Panagakis A, Despotidis M, Yfantopoulos J. The impact of the COVID-19 pandemic on surgical activities: a single-center experience and literature review. Cureus. 2022;14: e30785. doi: [10.7759/cureus.30785](https://doi.org/10.7759/cureus.30785). eCollection 2022 Oct.

26.	Vissio E, Falco EC, Collemi G, Borella F, Papotti M, Scarmozzino A, et al. Impact of COVID‐19 lockdown measures on oncological surgical activity: analysis of the surgical pathology caseload of a tertiary referral hospital in Northwestern Italy. J Surg Oncol. 2021;123: 24-31. doi: [10.1002/jso.26256](https://doi.org/10.1002/jso.26256).
